# Supplementary figures and images for: Characterizing urban landscapes using very-high resolution satellite imagery to predict Ae. albopictus larval presence probability in public spaces
Source: PLoS One. 2025 Nov 5;20(11):e0335794. doi: 10.1371/journal.pone.0335794 (PMC12588498; doi:10.1371/journal.pone.0335794)

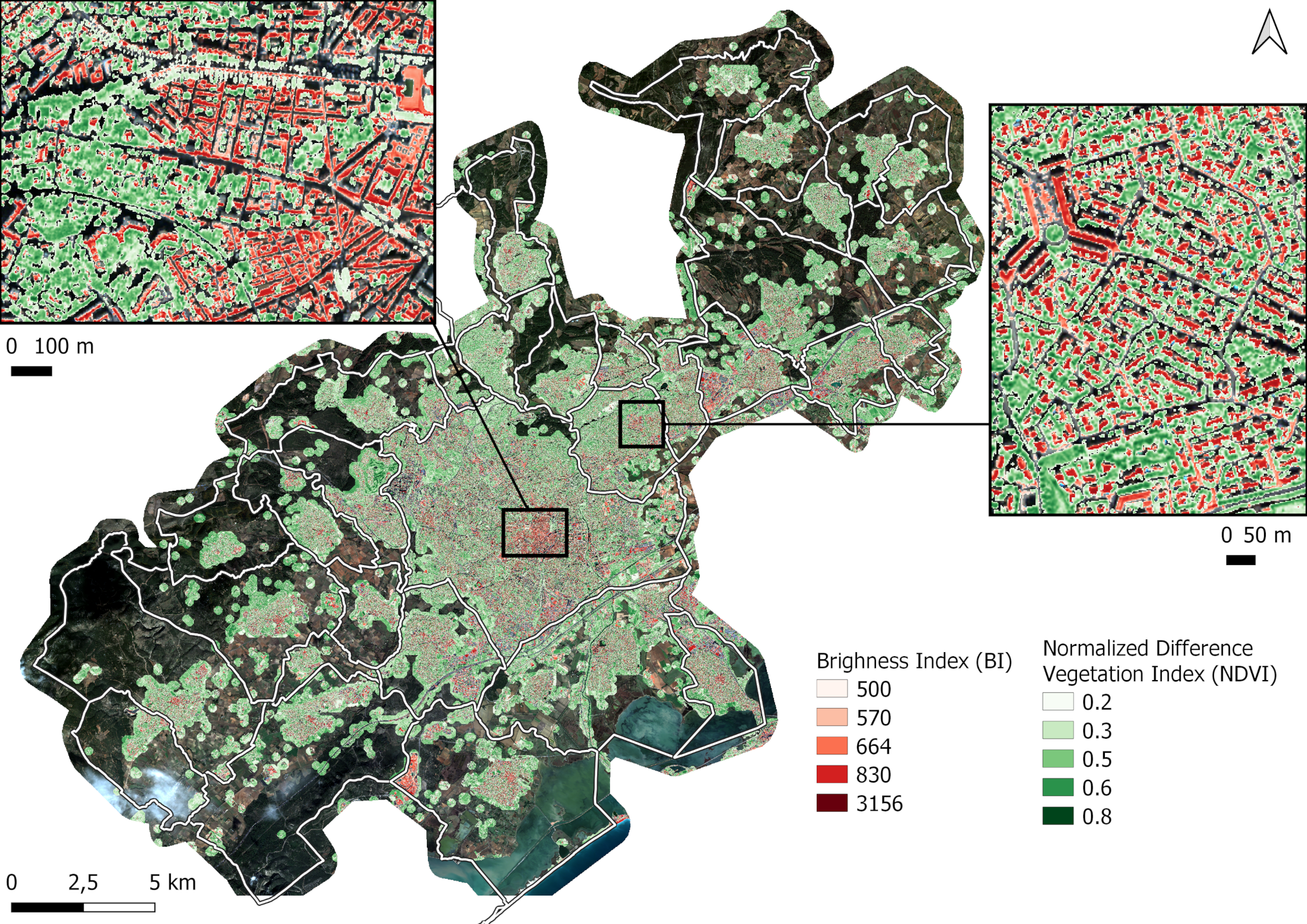

Supplement: S1 File — The top-left inset shows the dense city center of Montpellier, while the top-right one shows a residential area in a nearby municipality. (TIFF) [file pone.0335794.s001.tiff]

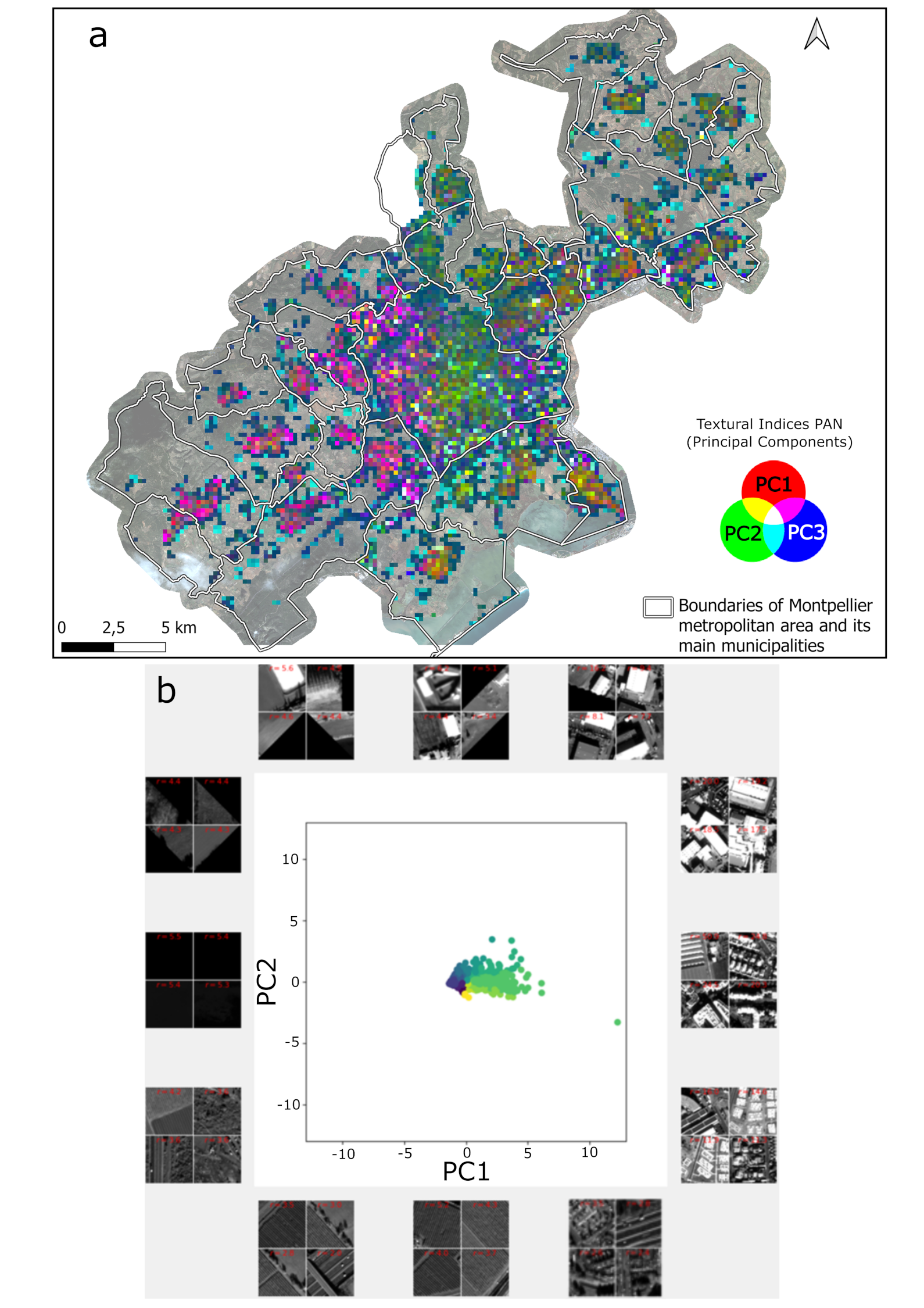

Supplement: S2 File — The colors correspond to the different angular sectors of point cloud individuals. Images subsets correspond to the analysis windows furthest from the axis origin, for each angular sector. (TIFF) [file pone.0335794.s002.tiff]

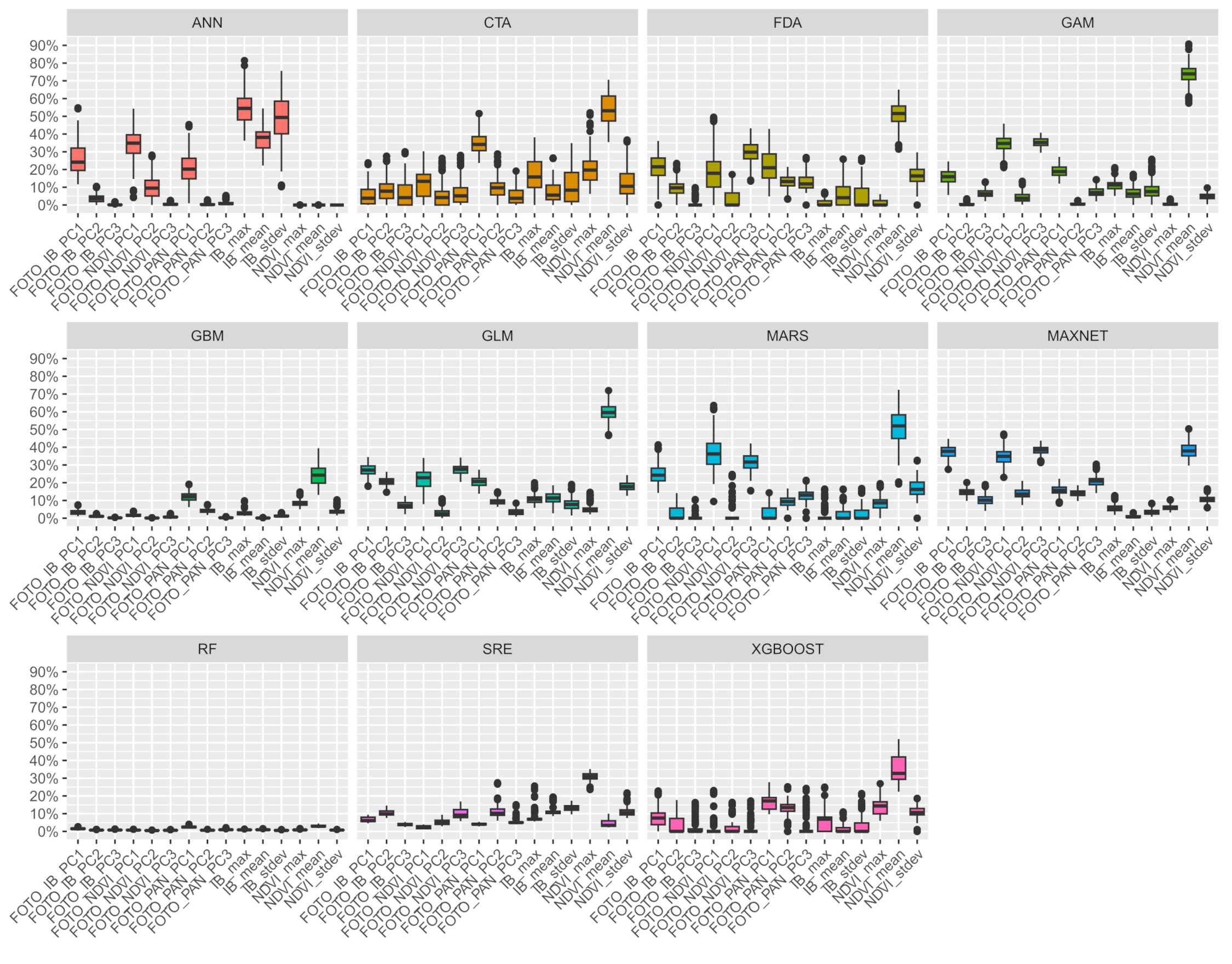

Supplement: S3 File — (TIFF) [file pone.0335794.s003.tiff]
