# Supplementary material for: Characterizing urban landscapes using very-high resolution satellite imagery to predict Ae. albopictus larval presence probability in public spaces
Source: PLoS One. 2025 Nov 5;20(11):e0335794. doi: 10.1371/journal.pone.0335794 (PMC12588498; doi:10.1371/journal.pone.0335794)
Supplement: Attachment — Submitted filename: PONE-D-25-16377 Revision.docx [file pone.0335794.s004.docx]

REVISION DE L ARTICLE:

Predicting Ae. albopictus larval presence probability in public spaces using very high- resolution satellite imagery

Minor concerns

Abstract:

“particularly in storm drains and telecom cable chambers in Montpellier, France. ·: Could you rephrase this sentence?

“evaluated “

What was evaluated ? Do you mean “their accuracy was evaluated”?

NVDI meaning: define the abbreviation in its first appearance in the text on line 35. Normalized Difference Vegetation Index?

42 ultimately contributing to improved public health outcomes in the face of
43 vector-borne disease threats.

Would write public health policies outcomes.

51. Ae. Albopictus ………..role as a primary vector for  diseases such as dengue, chikungunya, and Zika.

Would be relevant to mention that this is the case in Asia, not so much in America.

63. Ae. albopictus breeding sites - i.e., sites where
64. 65  mosquitoes lay eggs and where larvae develop - are known to be common in a wide range of artificial containers [9]

Rephrase.

76. Despite a presence of Ae. albopictus for two decades, mainland France does not experience regular  dengue outbreaks,
    77 unlike French tropical overseas regions, such as Martinique, French Guiana, and
    78 Reunion Island [14–16]

The phrase is confusing

Line 80 “Coupled with” “considering the” would be better suited

Line 107 “in the private domain “rather “private properties”

108 they typically represent the majority of known breeding sites [32,33]. However,

109  it is known that public spaces also account for the most breeding sites,

Contradictory sentence: the majority and the most are the same thing

Line 115 ¨analyzing¨ to be replaced by ¨analysis of the¨

Line 117

between species and their  environment, and predict where a species might ~~potentially~~ be found [35,36].

A prediction is probabilistic hence always with a grade of uncertainty.

Line 122 “enhance predictions” what aspect of the prediction would be enhanced?

Line 124 “SDMs can help to identify” rather “can help identify”

Line 129 “ in which extent “-> to which extent

Line 139 of 306,000 People

Line 144 However, there have also been autochthonous dengue cases TO However, autochthonous dengue cases have also been reported

Line 155. That is -> such as

Line 163 “then water and larvae “. Since the precondition is that there is presence of water, the only deduction is the presence of larvae.

Line 167 “and water does not drain properly from storm drains that tend to be poorly maintained. “ rather: storm drains tend to be poorly maintained impeaching the water to drain properly.

LINE 170 “On the contrary “ rather use “ conversely”.

LINE 172-175

Several types of breeding sites were inspected in public spaces: storm drain, open retention basins, telecom cable  chambers, urban pits, rural pits, underground waste garbage cans, and reservoir structures. In this study,  only storm drains (Fig. 1a)

Change to

Several types of public spaces breeding sites were inspected

Give the references for this asumption

LINE 179

We hence decided to exclude climatic TO Hence, we decided

Line 189 study area -> studied area

Line 217 Then, we created a colored composition of the

Coma

Line 241

“In other words, for a given cell, the greater the distance to a sampled cell is, the less the observations (samples) are representative of the specific environmental conditions of this cell, and the less a model built with the observations will be reliable.

The sentence should be split for clarity.

In short, machine learning allows the data to dictate the form of the model, whereas conventional statistics attempts to fit the data to an investigator-specified model.

Line 212

Window size of 101 pixels (i.e 202 meters) ..Precise the conversion, pixels are superficy unit unless aligned.

Table 1: broadening of the first column to avoid dividing panchromati C.

Line 293

The city center has less vegetation, as do certain commercial and industrial zones

¿is it “in contrast with” instead of “as do”

Line 320

(where no prior knowledge or complementary data is required)

Would rather use where no prior knowledge or complementary data are required.

Line 331

Montpellier being the main city center (purple), surrounded by peripheral secondary centers (pink), and the same type of urban gradients observed for each urban center

Sentence structure unclear

Fig. 3 clarify the figure legend and improve the letters readability of scales in the figure.

Line

Line 343 Fig. 4 caption: evidenced. Indicated could be more appropriated.

Line 426 “Regarding texture indices, the probability strongly increases when the first BI

426 component has values above 5, reaching values above 0.6” Unclear: what probability must be above 0.6?

Line 488 “inform”, would be “form” or shape more appropriate?

Line 506 “in relationship with Ae. albopictus breeding sites” would “ and associate it with Ae. albopictus breeding sites” be better?

Line 528 “In regions where dengue is present but not yet endemic, health concerns are less critical, leading to more limited resources for systematic vector surveillance, affecting in turn data availability and the volume of data needed to build robust predictive models.” Phrase should be split.

Line 531 would put frequent instead of ·regular”

Line 563 would write “therefor Our results help characterize the environment favorable to the presenceof positive breeding sites.” Instead of Our results thus help characterize the environment favorable to the presence of positive breeding sites.

Line 578 would write “This raises questions about the resources dedicated to data collection in the field” instead of This raises questions of the resources dedicated to data collection in the field

Line 594 that if water remains stagnant in poorly designed or poorly maintained breeding sites

Breeding sites or stagnant water urban structure? Otherwise would sound as the city do design and maintain breeding site.

Line 599 “Public breeding sites that drain well will not fill up with water during those two months and will not retain water, but, if there is a design flaw in some public breeding sites, water that will fill them up will partially stagnate and remain present throughout the winter if no manual  drainage is made, since the weather conditions will prevent evaporation.”

Split the phrase

Line 630 “It is thus necessary to continue implementing more efficient, and optimized vector surveillance and control strategies which could benefit from stronger links between operational and research sectors, particularly in order to question the transferability of knowledge, data, methods (e.g. on sampling design, landscape characterization, data analysis and modeling approaches) related to dengue-endemic areas.”

Phrase should be split
