# Supplementary material for: Characterizing urban landscapes using very-high resolution satellite imagery to predict Ae. albopictus larval presence probability in public spaces
Source: PLoS One. 2025 Nov 5;20(11):e0335794. doi: 10.1371/journal.pone.0335794 (PMC12588498; doi:10.1371/journal.pone.0335794)
Supplement: Attachment — Submitted filename: Response_to_reviewers.docx [file pone.0335794.s006.docx]

Response to reviewers

Ref: PONE-D-25-16377

Line numbers refer to the lines in “revised mansucript with track changes” document.

| **Editor comments** | **Authors responses** |
| --- | --- |
| 1) Authors must focus on determining larval presence probability. | The four comments of the Editor provided in this section of the change report seem to echo key concerns of the Reviewer 1 and have been answered in more details in the Reviewer 1 responses (and not repeated here in full to avoid duplication).  This first comment appears to come from a misunderstanding about the key contribution of our paper that is methodological in nature (see response made to Reviewer 1), hence explaining the strong focus on the method used to prepare the data for the model, instead of only presenting the model. To avoid different expectations that could have come from the original title, we have modified the title of the article: “*Characterizing urban landscapes using very-high resolution satellite imagery to predict Ae. albopictus larval presence probability in public spaces*”. To clarify the methodological nature of our contribution, we have modified the introduction by adding the following sentences (lines 130-135): *“This study places particular emphasis on urban landscape factors derived from very-high resolution imagery, as these variables are not only interesting inputs for the species distribution model, but also offer an opportunity to explore and better understand the complex relationships between urban landscapes and larval presence probability in public areas”* |
| 2) Authors must develop a model that accurately predicts mosquito larval presence. | This comment is confusing as we believe our paper indeed focused on the development and use of a model that accurately predicts the probability of larval presence. As now clarified in the Methods section (lines 169–170 and 173-175), the positivity of breeding sites was recorded during field surveys based on the presence of water combined with a favorable immediate urban context for *Aedes* development, as assessed by trained vector control agents. This operational definition reflects the surveillance practices currently implemented in temperate regions like ours, where arboviral risks are recent and survey protocols are adapted to operational constraints. We have acknowledged the limitations of our dataset in the Discussion section, particularly regarding the assumptions made by experts and the uneven spatial distribution of sampling. The Methods section describes the data used in the model and the model itself. The results section reports on the use of the model and its predictions. |
| 3) Please explain better the results of positive containers with water and ponds with larvae. | While the original dataset from EID-MED agents included surveys of breeding sites potentially associated with *Culex* species, our study specifically focuses on two types of *Aedes albopictus* breeding sites: storm drains and telecom cable chambers. These were selected because vector control experts identified them as particularly problematic and important in terms of *Aedes albopictus* larval presence in urban environments (also see response made to Reviewer 1 for more details). We have clarified the description of the entomological data in Section 2.2 of the revised manuscript. |
| 4) It is also recommended that temperature data be incorporated into the models. | We thank the Editor for this suggestion. More details on this have been provided in our response to Reviewer 1. Climatic variables were not included due to the large spatial resolution mismatch between existing climatic datasets and the very fine-scale of the spatial imagery being used. Indeed, the number of in-situ meteorological stations is limited in our area of study, and the coarse resolution of satellite-based climatic products is unsuitable for our objective of characterizing urban landscape features at a fine scale. Such products would only be relevant if we were conducting a study at a regional scale, as it has been done in other studies. Moreover, this study also chose to focus on urban landscape variables derived from very-high resolution imagery, a type of predictor that remains less frequently explored in the literature, to better understand the factors of *Aedes albopictus* larval presence in public spaces. |

| **Reviewers comments** | **Authors responses** |
| --- | --- |
| **Reviewer 1** |  |
| The article titled "Predicting Ae. albopictus Larval Presence Probability in Public Spaces Using Very High Resolution Satellite Imagery" by Claire Teillet et al. examines the influence of urban landscapes on the presence and distribution of Ae. albopictus larvae, particularly within two specific niches. The study evaluates environmental bias by analyzing the representativeness of sampled reproduction sites across various urban landscapes. The authors employ Species Distribution Models (SDMs) to predict larval presence in the study area, identifying the Normalized Difference Vegetation Index (NDVI) as the most influential variable, with large vegetation patches increasing the likelihood of larval presence. This research is significant due to the rising incidence of dengue cases in non-endemic regions, such as the study area. Aerial imagery has previously been utilized as a tool for monitoring and predicting larval and adult mosquito development sites. The study's relevance lies in applying these tools in areas without sustained dengue endemicity, specifically focusing on public mosquito development sites. | N/A |
| However, the article's emphasis on image processing and model development somewhat overshadows the primary objective of determining larval presence probability, which is only addressed between pages 432-449 in the results section. It is recommended that the authors reduce the specialized content and concentrate on developing a model that accurately predicts mosquito larval presence. | The key innovation brought by our paper is methodological in nature, the main objective (stated on line 137) being “*This paper* ***proposes a novel approach*** *to help understand how urban landscape structure and composition affect positive breeding sites of Ae. albopictus in the public domain. It* ***aims to determine to which extent landscape factors, derived from remote sensing images****, can help explain the presence of positive Aedes breeding sites in public areas and to use these factors to predict the presence probability of such sites in the Montpellier metropolitan area, southern France, where the presence of Ae. Albopictus has increased over the last two decades*”.  While predicting larval presence probability is an important outcome of this work, we believe it is essential to ensure transparency regarding the assumptions allowing to exploit the data provided by the vector control services and the production of environmental variables used as inputs in the prediction model, and to discuss the potential environmental biases associated with the surveillance data we used for modeling, as these directly affect the reliability and performance of the presence probability predictions. We have modified the title of the article accordingly to better reflect the objective of the study, now reading “*Characterizing urban landscapes using very-high resolution satellite imagery to predict Ae. albopictus larval presence probability in public spaces*”. |
| Additionally, the authors should provide more comprehensive data, as the entomological data used to monitor storm drains and telecom cable chambers lacks clarity regarding larval positivity, which is a crucial inclusion criterion. It is assumed that a census of such containers exists throughout the city, but it is unclear if this correlates with the risk areas depicted in Figure 9. | We thank the reviewer for this comment. Entomological data were collected based on the operational capacities and priorities of the EID-MED services (now clarified on line 164).  We have also clarified the definition of breeding sites data in the manuscript (lines 169-170, 173–175) to explain that, during winter surveys, a container was classified as “positive” based on the presence of stagnant water combined with a favorable urban context, according to vector control agents’ expertise.  Additionally, we specified that this dataset results from exploratory surveys from EID-MED services with an operational purpose rather than a systematic census (lines 193-194).  We acknowledge the limitations of our dataset and have discussed them in Section 4.5, specifically on the strong assumptions made by experts (line 622) and on the uneven spatial distribution of the sampling (line 650). It is important to understand that survey protocols in the study region are not (yet) as thoroughly done as in many other places (e.g., tropical countries) as this region did not historically have arboviruses. This specific context of emerging risks brings a very interesting angle to the study and applies to many countries or regions where those viruses are emerging. This point has now been added and highlighted in the manuscript on lines 669-679: *“More broadly, the specific context of emerging arboviral risks in this region means that entomological surveillance protocols have traditionally been less intensive than in endemic tropical areas, which partly explains current limitations in data coverage and sampling diversity. This context of emerging risk in a temperate region adds a particularly valuable perspective to this study, as it reflects the challenges encountered by many countries or regions now facing the emergence of these diseases. Importantly, this study highlights the relevance and feasibility of developing spatial models using data constrained by operational realities and specific contexts. Understanding these operational and logistical constraints is essential to interpreting the limitations of current datasets and to improving surveillance and control strategies.”* |
| Furthermore, the resolution of the satellite images should be clarified to determine if these potential larval niches and their relationship with surrounding vegetation can be effectively monitored. | We are unclear on this comment as the resolution of satellite images was clearly stated in the original manuscript, with the text stating, “*Two very high-resolution Pléiades satellite images of the study area acquired on November 2 and 6, 2021 were used in the study. Orthorectified Pléiades images include a 50 cm resolution panchromatic band and four 2 m resolution multispectral bands (blue, red, green, near-infrared).*” (page 76, line 214)  While individual small containers, storm drains, or telecom chambers cannot obviously be directly detected with satellite images, their environment can be effectively monitored, being often used in the literature to support *Aedes albopictus* habitat modeling. Recent studies (Machault et al., 2017; Marti et al., 2020; Bailly et al., 2021; Teillet et al, 2024) have, for instance, shown the potential of very high-resolution imagery for improving vector habitat risk mapping in urban environments. We clarified this aspect on lines 219-221. |
| It is also important to ascertain whether these sites are relevant for the development of Aedes albopictus or other mosquito species, such as Culex spp., which could be correlated with the positivity index and presence of these species in the deposits. The results of the monitoring remain unclear to me, particularly regarding the presence of positive containers with water and ponds with larvae. These data are crucial as they directly indicate the model's success and its correlation with positive deposits of mosquito larvae. | Although the original dataset included breeding sites associated with *Culex* spp., we specified that this study focuses exclusively on breeding sites associated with *Aedes albopictus* (lines 190). For this study, we specifically focused on **storm drains and telecom cable chambers**, as these two types of artificial containers are well-documented breeding sites for *Aedes albopictus* (as explained and referenced on lines 182-184 and 189-191). Vector control services (EID-MED) expertise and surveillance have shown that these two container types play an important and problematic role in the presence of *Aedes albopictus* larvae in urban areas, which also justifies our decision to restrict the analysis to these specific breeding sites.  To address the second part of the comment, we have clarified in the revised manuscript (lines 169-170,173–175) how containers were classified as positive. We acknowledge the exploratory nature and limitations of this dataset and have explicitly discussed these aspects in Section 4.5, including the expert assumptions made when classifying breeding sites as positive (lines 623-649). |
| How frequently have such positive containers for Aedes albopictus larvae been identified? | On-site inspections were conducted between November 2021 and March 2022. Information on the precise dates or intervals between inspections during this period was unfortunately not made available by the agency that provided the data. |
| Furthermore, it is noted that other parameters, such as temperature, have not been considered. However, the authors themselves mention in lines 585-588 that "An unusual aspect of the entomological dataset used is that data on breeding sites and their positivity to larvae were collected in the metropolitan area during the winter period (i.e., November to March), which is not the period during which larvae are more likely to be found due to predominantly unfavorable temperature conditions." Therefore, incorporating temperature data into the models may be advisable. Although I understand that MaxEnt utilizes temperature data, it appears that the authors have neither collected nor mentioned such data. Climate parameters, such as temperature, as the authors have indicated, facilitate larval growth. I hypothesize that these types of containers are located in various areas with specific microclimatic conditions; can this be documented or correlated with larval growth? I contend that the rationale for excluding climate data is insufficiently substantiated, and I suggest that the authors emphasize or describe climatic conditions that could correlate with the likelihood of finding larvae. | We acknowledge that climatic parameters, particularly temperature and precipitation, are known to influence mosquito larval development and habitat suitability. In our study, temperature and precipitation data were not integrated into the analysis (even in the Maxent model and ensemble model) for several reasons:  (i) our specific objective, as explained in the manuscript was to “*to determine to which extent landscape factors, derived from remote sensing images, can help explain the presence of positive Aedes breeding sites in public areas and to use these factors to predict the presence probability of such sites in the Montpellier*” (line 138). The focus was placed on urban landscape characteristics rather than climatic variables. The relationship between breeding sites and urban landscape patterns remains underexplored, especially using very high spatial resolution imagery, and our study aims to address this gap.  (ii) there is a large spatial resolution mismatch between existing climatic datasets and the very fine-scale of the spatial imagery being used. Indeed, the number of in-situ meteorological stations is limited in our area of study, and the coarse resolution of satellite-based climatic products is unsuitable for our objective of characterizing urban landscape features at fine scale. Such products would only be relevant if we were conducting a study at regional scale as it has been done in other studies. Moreover, at the scale of the metropolitan area of Montpellier, there is no strong microclimatic variability that would justify the integration of coarse-resolution climate data into fine-scale intra-urban modeling.  (iii) additionally, while the inspections were conducted during an unfavourable season for *Aedes albopictus* larval development, our choice to focus on specific types of breeding sites (storm drains and telecom cable chambers) was based on strong field expertise and the assumption that water tends to persist in these containers throughout the year, irrespective of seasonal variations. These structures are known to be perennial breeding sites in urban environments, even during colder months, which justifies their selection for spatial modelling. We prioritized environmental and landscape variables over climatic ones, as the persistence of water in these breeding sites is more related to urban infrastructure characteristics than to microclimatic fluctuations.  We have clarified these points and completed with references in the revised manuscript (lines 197-207). |
| In the conclusions, between lines 654-656, it is stated that "These predictive maps can be useful for optimizing the allocation of resources, ensuring that vector control efforts are concentrated in the most critical areas to reduce mosquito density and thus limit the spread of vector-borne diseases." This prompts me to inquire about the current distribution of the species in the study area. While there are cases of native dengue, the known distribution of Aedes albopictus has not been addressed. If this data were available, we could discuss the current distribution and the potential implications of other risky niches. | Although general distribution maps of *Aedes albopictus* do exist at regional levels (Roche et al., 2018), there is currently no existing fine-scale map for Montpellier. Our study contributes to addressing this gap by identifying landscape variables and areas most likely to harbour potential positive breeding sites, based on environmental predictors derived from very high-resolution imagery. These predictive maps do not directly represent adult mosquito distribution. However, they offer a complementary tool to entomological surveillance by highlighting areas with high environmental probabilities for presence of breeding sites, which is determinant for adult population density and could inform vector control operations at very fine scale. |
| Additionally, it is important to note that vector density is not necessarily indicative of vector competence, and this should also be included in the discussion. | This is very true, and we now nuance this point in the discussion on lines 605-608. |
| **Reviewer 2** |  |
| Abstract:  “particularly in storm drains and telecom cable chambers in Montpellier, France. ·: Could you rephrase this sentence? | Corrected on line 32. |
| “evaluated “  What was evaluated ? Do you mean “their accuracy was evaluated”? | Corrected on line 36. |
| NVDI meaning: define the abbreviation in its first appearance in the text on line  35. Normalized Difference Vegetation Index? | Corrected on line 39. |
| 42 ultimately contributing to improved public health outcomes in the face of  43 vector-borne disease threats.  Would write public health policies outcomes. | Corrected on line 47. |
| 51. Ae. Albopictus ………..role as a primary vector for diseases such as dengue, chikungunya, and Zika.  Would be relevant to mention that this is the case in Asia, not so much in America. | Corrected on line 56. |
| 63. Ae. albopictus breeding sites - i.e., sites where  64. 65 mosquitoes lay eggs and where larvae develop - are known to be common in a wide range of artificial containers [9]  Rephrase. | Corrected on line 70. |
| 76. Despite a presence of Ae. albopictus for two decades, mainland France does not experience regular dengue outbreaks,  77 unlike French tropical overseas regions, such as Martinique, French Guiana, and  78 Reunion Island [14–16]  The phrase is confusing | Corrected on line 81. |
| Line 80 “Coupled with” “considering the” would be better suited | Corrected on line 86. |
| Line 107 “in the private domain “rather “private properties”  108 they typically represent the majority of known breeding sites [32,33]. However,  109 it is known that public spaces also account for the most breeding sites,  Contradictory sentence: the majority and the most are the same thing | We clarified this sentence (lines 113-115). |
| Line 115 ¨analyzing¨ to be replaced by ¨analysis of the¨ | Corrected on line 120. |
| Line 117  between species and their environment, and predict where a species might potentially be found [35,36].  A prediction is probabilistic hence always with a grade of uncertainty. | We totally agree that species distribution models provide probabilistic predictions that inherently include a degree of uncertainty.  The model was evaluated by means of classical performance metrics such as the Area Under the Curve (AUC) of a Receiver Operating Characteristic (ROC) curve and the True Skill Statistic (TSS)  Imprecision and uncertainty were also discussed by questioning the representativity of the sampling in section 4.4 of the manuscript, especially on lines |
| Line 122 “enhance predictions” what aspect of the prediction would be enhanced? | We clarified this sentence (lines 127-128). |
| Line 124 “SDMs can help to identify” rather “can help identify”  Line 129 “ in which extent “-> to which extent  Line 139 of 306,000 People | Corrected on lines 130,139,149. |
| Line 144 However, there have also been autochthonous dengue cases TO However, autochthonous dengue cases have also been reported | Corrected on line 154. |
| Line 155. That is -> such as | Corrected on line 166. |
| Line 163 “then water and larvae “. Since the precondition is that there is presence of water, the only deduction is the presence of larvae. | We clarified this on lines 169-170. |
| Line 167 “and water does not drain properly from storm drains that tend to be poorly maintained. “ rather: storm drains tend to be poorly maintained impeaching the water to drain properly. | Corrected on line 181. |
| LINE 170 “On the contrary “ rather use “ conversely”. | Corrected on line 185. |
| LINE 172-175  Several types of breeding sites were inspected in public spaces: storm drain, open retention basins, telecom cable chambers, urban pits, rural pits, underground waste garbage cans, and reservoir structures. In this study, only storm drains (Fig. 1a)  Change to  Several types of public spaces breeding sites were inspected  Give the references for this asumption | We corrected the sentence line 188. We selected these specific types of breeding sites in this study based both on the operational expertise of EID-MED and on recommendations from the literature (lines 182–190). |
| LINE 179  We hence decided to exclude climatic TO Hence, we decided  Line 189 study area -> studied area  Line 217 Then, we created a colored composition of the  Coma | Corrected on lines 205, 210, 246. |
| Line 241  “In other words, for a given cell, the greater the distance to a sampled cell is, the less the observations (samples) are representative of the specific environmental conditions of this cell, and the less a model built with the observations will be reliable.  The sentence should be split for clarity. | Corrected on line 273. |
| In short, machine learning allows the data to dictate the form of the model, whereas conventional statistics attempts to fit the data to an investigator-specified model. | We generally agree with the reviewer on the difference between machine learning and statistical models. |
| Line 212  Window size of 101 pixels (i.e 202 meters) ..Precise the conversion, pixels are superficy unit unless aligned. | Corrected on line 240. |
| Table 1: broadening of the first column to avoid dividing panchromati C. | Corrected on Table 1. |
| Line 293  The city center has less vegetation, as do certain commercial and industrial zones  ¿is it “in contrast with” instead of “as do” | We intended to express that both the city center and certain commercial and industrial zones have similarly low levels of vegetation. Therefore, we believe that *as do* is appropriate here, as it highlights this similarity rather than a contrast (line 325). |
| Line 320  (where no prior knowledge or complementary data is required)  Would rather use where no prior knowledge or complementary data are required. | Corrected on line 352. |
| Line 331  Montpellier being the main city center (purple), surrounded by peripheral secondary centers (pink), and the same type of urban gradients observed for each urban center  Sentence structure unclear | We clarified the sentence line 362. |
| Fig. 3 clarify the figure legend and improve the letters readability of scales in the figure. | Corrected on Fig 3. |
| Line 343 Fig. 4 caption: evidenced. Indicated could be more appropriated. | Corrected on line 376. |
| Line 426 “Regarding texture indices, the probability strongly increases when the first BI  426 component has values above 5, reaching values above 0.6” Unclear: what probability must be above 0.6? | To clarify, we meant that the probability of a positive breeding site exceeds 0.4 when the value of the first BI component is greater than 5. We have rephrased the sentence accordingly to avoid any ambiguity (line 458). |
| Line 488 “inform”, would be “form” or shape more appropriate? | We clarify the sentence on line 522. |
| Line 506 “in relationship with Ae. albopictus breeding sites” would “ and associate it with Ae. albopictus breeding sites” be better? | Corrected on line 540. |
| Line 528 “In regions where dengue is present but not yet endemic, health concerns are less critical, leading to more limited resources for systematic vector surveillance, affecting in turn data availability and the volume of data needed to build robust predictive models.” Phrase should be split. | We clarified these sentences by splitting them - see lines 562-564. |
| Line 531 would put frequent instead of ·regular” | Corrected on line 567. |
| Line 563 would write “therefore Our results help characterize the environment favorable to the presence of positive breeding sites.” Instead of Our results thus help characterize the environment favorable to the presence of positive breeding sites. | Corrected on line 598. |
| Line 578 would write “This raises questions about the resources dedicated to data collection in the field” instead of This raises questions of the resources dedicated to data collection in the field | Corrected on line 616. |
| Line 594 that if water remains stagnant in poorly designed or poorly maintained breeding sites  Breeding sites or stagnant water urban structure? Otherwise would sound as the city do design and maintain breeding site. | Corrected on lines 630-633. |
| Line 599 “Public breeding sites that drain well will not fill up with water during those two months and will not retain water, but, if there is a design flaw in some public breeding sites, water that will fill them up will partially stagnate and remain present throughout the winter if no manual drainage is made, since the weather conditions will prevent evaporation.”  Split the phrase | We split these sentences, see line 638. |
| Line 630 “It is thus necessary to continue implementing more efficient, and optimized vector surveillance and control strategies which could benefit from stronger links between operational and research sectors, particularly in order to question the transferability of knowledge, data, methods (e.g. on sampling design, landscape characterization, data analysis and modeling approaches) related to dengue-endemic areas.”  Phrase should be split | We split these sentences, see line 667. |
